# Written on the Skin: Participants’ Perspectives on Scarring, Discolouration and Implant Site Reactions Experienced During a Phase I Clinical Trial for a Subdermal HIV Prevention Implant in KwaZulu-Natal, South Africa

**DOI:** 10.1007/s10461-026-05113-6

**Published:** 2026-04-17

**Authors:** Emma Durden, Eliza Govender, Aphiwe Dlokweni, Nqobile Ngubane, Nqobile Myeni, Ishana Harkoo, Leila E. Mansoor, Quarraisha Abdool Karim, Salim S. Abdool Karim, Tanuja N. Gengiah

**Affiliations:** 1 Culture, Communication and Media Studies, College of Humanities, University of KwaZulu-Natal, Durban, South Africa; 2 Centre for the AIDS Programme of Research in South Africa, University of KwaZulu-Natal, Durban, South Africa; 3 Department of Epidemiology, Columbia University Mailman School of Public Health, New York City, USA

**Keywords:** Subdermal implant, PrEP, scarring, implant site reactions.

## Abstract

To address challenges associated with the uptake of daily oral pre-exposure prophylaxis (PrEP) by young women at high risk of acquiring HIV, a range of novel, long-acting, slow-release products are being developed and clinically evaluated. One such technology is an annual implant of tenofovir alafenamide (TAF). This study explores the perspectives of participants from a first-in-human trial of an annual TAF implant to inform considerations for future PrEP implant innovation. The study, conducted in Durban, South Africa between 2022 and 2023, included 29 in-depth interviews (IDIs) and three focus group discussions (FGDs) with 18 participants. All participants experienced some form of implant site reaction (ISR). In some instances, these were minor, and in others, the implant removal process left longer-lasting and more severe skin changes. Participants’ perceptions of these ISRs were influenced by concerns about their appearance, social stigma, and associations between scars and gender-based violence. Mild skin changes were deemed acceptable, while severe scarring and discolouration caused concerns about attracting negative attention. Few participants expressed concerns about their own scars, however, visible scars which might be mistaken for a contraceptive implant caused concern. Despite the ISRs experienced, there was positive feedback for this type of technology, and many considered that other women would be interested in adopting a sub-dermal implant should it be effective in preventing HIV. Continued enhancements of next generation implants to enhance drug release rates should consider how to minimise the severity and visibility of ISRs.

## Introduction

Adolescent girls and young women (15–24 years old) continue to bear the brunt of new HIV acquisition, accounting for approximately 63% of all new acquisitions in Sub-Saharan Africa [1], most often from sexual partners who are eight or more years older than them [2, 3]. Daily, oral tenofovir is currently a widely available safe and effective pre-exposure prophylaxis (PrEP) for HIV prevention that provides some agency to young women in these age-sex disparate relationships. However, uptake and persistent use remain low and are influenced by perceptions of risk and the personal, relational and economic costs that are associated with access and use of these interventions [4].

To overcome uptake and adherence challenges with oral PrEP, studies of longer acting agents including annual implants are under clinical evaluation. The CAPRISA 018 first-in-human trial assessed the safety, acceptability, tolerability, and pharmacokinetics (PKs) of a tenofovir alafenamide (TAF) free-base subdermal implant formulation and was undertaken in Kwa-Zulu Natal, South Africa between 2020 and 2022 [5].

The CAPRISA 097 prospective cohort study was undertaken among consenting CAPRISA 018 study participants to monitor long-term sequelae and the duration to resolution of local side-effects after implant removal and study exit in CAPRISA 018 study participants. An additional qualitative component was added to the CAPRISA 097 study to understand the experiences of the trial participants during and after the trial to inform product enhancement considerations for future product development.

Understanding the end-user product acceptability of new technologies based on real world experience of use can provide essential guidance for product development that could enhance acceptance, uptake and tailored demand creation strategies [6, 7].

This paper explored the perspectives of previous annual TAF implant trial participants enrolled in the CAPRISA 097 prospective cohort study, focused on the implant insertion site reactions (ISRs) and post-implant removal local reactions, and how these influenced the acceptability of future use of HIV PrEP implants.

## Methods

This study was conducted between October 2022 and October 2023. The study population comprised all 36 participants enrolled in the parent CAPRISA 018 clinical trial. Most participants, 35/36 (97%), consented to enrol into the CAPRISA 097 study including the qualitative in-depth interviews (IDIs) and focus group discussions (FGDs). All study participants provided written informed consent and ethics approval was obtained from the University of KwaZulu-Natal Biomedical Research Ethics Committee (Reference: BREC/00004541/2022). Beneficence and non-maleficence were ensured by minimising any potential discomfort to participants; autonomy was protected through informed consent; justice was addressed by fair recruitment; and research integrity was maintained through transparent reporting and reflexive analysis.

An independent team of researchers from the University of KwaZulu Natal (UKZN), who were not involved in the CAPRISA 018 clinical trial, conducted the IDIs and FGDs, and data analysis was carried out by an analyst who had no direct contact with the participants. For the IDIs, a pre-tested semi-structured interview guide was administered in English or isiZulu, as per the participant’s preference. Questions related to their enrolment in the trial, their experience with the insertion, removal, and living with the implant, and their overall experience of the trial.

In addition to open-ended questions that explored their trial experiences, an audience response system (ARS) tool was utilised during the FGDs, allowing participants to submit responses to questions using handheld devices, facilitating immediate anonymised data capture and quantification of responses. In addition, anonymised photographs of mild, moderate and severe scarring and hyperpigmentation experienced by participants was shown during FGDs and participants were asked to reflect on their acceptability.

With permission from each participant, the IDIs and FGDs were recorded, and verbatim transcriptions developed and translated into English for analysis.

Following a pragmatic research paradigm, this study employed both reflexive thematic analysis (RTA) and frequency counting to analyse data. Pragmatism supports the use of multiple methods and modes of inquiry, enabling researchers to select approaches best suited to address the research question [8].

The analyst, a health communications scholar with a background in HIV prevention, used RTA [9, 10] to identify patterns of shared meaning in the transcripts. Coding was guided by the interview and FGD questions while remaining open to patterns emerging from the data. NVivo 14 was used to organise codes and illustrative quotes, but all interpretive decisions were made by the analyst, with further engagement of these decisions by the authors. Reflexive engagement involved repeatedly reviewing the data and considering how the analyst’s perspectives influenced coding and interpretation, ensuring that themes captured the participants’ shared experiences rather than the analyst’s assumptions.

To complement the RTA, we recorded recurring themes and patterns in interview participants’ responses using an Excel spreadsheet. This allowed us to note the prevalence of perspectives within the participant group. Although the number of participants was small (*n* = 29), it represented a substantial proportion of the total population. The counts were used to illustrate patterns rather than to support statistical generalisation, consistent with the exploratory and interpretive aims of the study.

Quotes used in this paper are attributed to pseudonyms chosen by the participants in the IDIs and FGDs.

## Results

All participants were Black, African, females, with a median (IQR) age 26 (21.5–29.5) years. Rich detailed data had been collected after interviews with 29 participants for the IDIs; and 18 of those participants also took part in the FGDs. We conducted three FGDs of six participants each, with one FGD comprising participants who had one implant inserted, one with participants who had two implants inserted and one with participants who had early implant removal.

Participants shared their experiences of local skin reactions after receiving the implant, living with the implant inserted under their skin, having it removed, and their feelings about any resultant scars and discoloration. These experiences, and viewing the scars and skin discoloration that some other participants experienced, led to varied reflections about the value of the implant versus its potential drawbacks.

The descriptive headings in the data presentation below correspond to the main areas of inquiry from the interview and FGD guides, to facilitate clarity and transparency for readers. In line with RTA, the analysis focuses on patterns of shared meaning across participants’ accounts, with a summary table (Table 1) showing how each heading relates to an analytic theme.

**Table 1:** Descriptive headings and associated reflexive themes

Headings	Reflexive themes
Implant visibility	Implant visibility was considered a threat to social comfort and discretion, linked to fears of unwanted attention, stigma, and potential danger.
Implant site reactions (ISRs)	ISRs disrupted participants’ sense of bodily comfort and produced some anxiety even when reactions were temporary.
Scarring	Scarring was largely normalised as ordinary and acceptable, minimising personal concern despite its association with other medical procedures.
Degrees of scarring	Acceptability of scarring varied, with severe or conspicuous scars perceived as socially and personally troubling.
Hyperpigmentation	Hyperpigmentation was perceived as socially threatening, as participants associated it with signs of physical abuse.
Prior warning of potential skin reactions	Transparency about potential adverse effects was weighed against the potential emotional or social harm to other women.
Considering the overall value of the TAF implant	Participants balanced potential skin changes against perceived health and practical benefits, interpreting the overall value of the implant through this trade-off.

### Implant Insertion and Visibility

The visibility of the implant, often because of short-term implant site reactions (ISRs) or the procedure itself, was considered a threat to social comfort and discretion, raising concerns for some participants about unwanted attention, stigma, and potential danger. Participants reported that immediate visible ISRs, tenderness, bruising and swelling, were not long-lasting and did not have a significant impact on how they felt about the implant.

*I only had that for a day and the next day I was alright*,* I had that discomfort that something had happened here*,* but after that I didn’t have anything.* (Nto, IDI)

*When a lump appeared here*,* I was a little regretful*,* but it was small and went away.* (Sbani, IDI)

For most participants, the implant was not visible on their arm once the initial swelling had worn down after insertion, and they appreciated this lack of visibility, particularly as it related to their own sense of privacy and not having to explain the implant to others.

*It was right that it’s not visible*,* because sometimes you don’t need to keep explaining to people.* (Nto, IDI)

For some participants, it was not the visibility of the implant itself that was a concern, but the bandage placed on their arm after the insertion, and the swelling or other side-effects that might draw attention to the site, particularly if they were touching the implant site.

*When you are walking in your neighbourhood*,* and you find that people can see it because its swollen. Now: ‘Oh you have inserted an implant*,* what happened?’ And all that stuff… They could see.* (Thando, IDI)

*The only time one could see something was when I was touching it or fiddling with it.* (Luh, IDI)

While most participants explained that their implant had not been visible, the thought of the implant being visible to others was disturbing to some.

*Everyone will see that you have now inserted an implant. It is better if it is hidden*,* and you are the only one that knows about it … There is no need for the whole community to see that you have inserted an implant.* (Sbani, IDI)

While concerns regarding a lack of privacy or the unwelcome questions and negative comments worried some participants, others were more confident in their ability to field questions and discuss the TAF implant, as contraceptive implants were not unusual amongst women in their community.

*I don’t have a problem with what people say. People will always talk anyway and ask you what is it that you have inserted. I can explain to them what it is that I have inserted*,* but I don’t mind if it’s being seen that I have inserted an implant*,* because it is something that is usually inserted*. (Apricot, IDI)

Beyond curious onlookers, one participant expressed a concern that had the implant been visible, it would have put her in danger:

*I felt that it would be obvious that the implant is on my arm. Out of the fear of the druggies. With the stories I heard that they remove implants so they can smoke the hormone that is inside*,* so that was my only worry.* (Yolanda, IDI)

This reflection reveals that beyond embarrassment, stigma or social discomfort, participants were conscious that their safety could be compromised if the implant were visible.

### Experience of Implant Site Reactions

Experiences of ISRs varied across participants, but even when short-lived, these reactions generated some anxiety, while longer-lasting reactions caused physical and emotional distress and, in some cases, led participants to request implant removal.

Participants noted that they had been advised of potential side-effects of the implant as a new technology, were cognisant of the need to report any side-effects to the clinical staff, and knew that they could get treatment for these.

*The side effect wasn’t bad; it was just an itchy lump that wanted me to scratch it. So*,* I avoided scratching it because I was running away from the fact that something might happen*,* it might burst. But then I came to the clinic and told them. They gave me something to apply*,* and it ended.* (Mimi, IDI)

While most participants reported that their ISRs were short-lived; some reported having longer-lasting reactions which caused them physical and emotional distress.

*What also scared me were the ugly marks that it was going to leave on my body. A body filled with dark marks is unappealing*,* so that is what scared me the most.* (Sky, IDI)

In extreme cases, ongoing or gradually worsening side-effects drove some participants to ask for an early removal of the implant.

*I was tired of this. I came back*,* and it was the same thing*,* because they came and gave me this cream. It only went down for a while and then it started again*,* so I didn’t know*,* maybe an infection or something was starting. So I just said that I should take it out straight away.* (Apple, IDI)

Participants who requested the early removals expressed disappointment, noting that they had wanted to see how the product would work.

*It was unfortunate to remove it*,* but I had to because I could see the negative side-effects*,* and no one wanted to wait and see what they would advance to. Hence*,* I came and asked that it be removed.* (Asanda, IDI)

While some participants opted for early removal of the implant due to their ISRs, most opted to continue with the implant, driven by their curiosity to witness the final results of the trial.

*I had doubts about the implant after having the side-effects and seeing things that I didn’t understand. I felt like removing it*,* but at the same time I wanted to see the results*. (Pinky, IDI)

### Concerns About Scars

Scarring resulting from implant insertion or removal, or the ISRs, was generally normalised by participants, with most describing scars as minor, commonplace, and not personally troubling.

In individual interviews, 24 of the 29 participants (83%) reported that they were not concerned about their own scars, noting that the scar was small, and most people’s bodies had scars, therefore the TAF implant scars would not be particularly noticeable.

Many explained how they were not affected or troubled by the minor scarring.

*It doesn’t scare me because it is like it was a bump. Even though I have a scar*,* I don’t have a problem because it doesn’t disturb me. It doesn’t hurt*,* and it doesn’t grow.* (Strawberry, IDI)

*Even with this*,* you won’t tell that something has been done*,* it seems as if I was scratched. So*,* the scars are not a problem*,* I’m not stressed about them.* (Nto, IDI)

Some noted that they might be more concerned about the scars if they were more prominent.

*I don’t have a problem with it*,* shame*,* because its hidden. If it was in a visible place … I would feel bad.* (Red, IDI)

Several participants commented that if the scar was visible, other people might think it was related to a vaccination or having a contraceptive implant. The thought that others would assume they had a contraceptive implant was concerning for some participants.

*I wish somehow that there could be something we receive after we have removed it to remove the marks*,* because it’s obvious they are from the implant. And other people don’t know*,* they know the implant is to prevent pregnancy only.* (Apple, IDI)

The five participants (17%) who were concerned about their own scarring reported that the removal process had been difficult for them, and resulted in visible and unsightly scars, which prompted unwanted curiosity from others. In one case, this scarring had affected the participants’ lifestyle, her manner of dress and her confidence.

*It doesn’t sit well with me at all*,* because now I must wear things with sleeves*,* I’m not used to that… After this implant incident I don’t like wearing revealing things like before*,* because when I wear something that reveals the scar*,* people ask me what happened*,* and I have to keep explaining… My confidence became low because now I can’t wear dresses with short sleeves until I am used to the scar.* (Yellow, IDI)

### Degrees of Scarring

Participants’ views on scarring varied with severity. While minor marks were generally tolerated, more severe or conspicuous scars were considered undesirable due to potential social attention and the perceived impact on the arm’s appearance, though some participants expected severe scars to become less noticeable over time.

Severe scarring (presented in anonymised photographs from the clinical trial) was considered unacceptable by 14 of the 18 FGD participants (78%), and they noted that they would not like this scarring themselves, and the unnecessary attention it would attract; suggesting it would deter people from implant use.

*I don’t see anyone agreeing to have an implant after seeing you with that scar. No person wants to draw negative attention to themselves and their body.* (Zahara, FGD 1)

Four participants (22%) in the FGDs, however, felt that the severe scarring shown was not an insurmountable problem, noting that it would likely fade with time.

Participants were almost equal in their acceptance or rejection of moderate scarring, noting that their own scars were similar, would fade in time, or resembled a scar from a minor injury or sore. However, some participants felt that even moderate scarring drew too much attention and would be difficult to explain and hard to live with.

*We can see that hole in the arm ruining the entire arm. You had an implant*,* yes*,* but this is unacceptable. That woman’s arm is ruined*,* shame.* (Nonku, FGD 3)

*I despise the fact that with that scar*,* you will have to keep explaining that it is not a contraceptive implant. It is to prevent HIV*,* and people will say there is no such*,* I’m lying.* (Zahara, FGD 1)

*Because of this*,* you can’t dress the way you like. You’re controlled by the scar. I refuse. That is too much for one to bear.* (Participant, FGD 3)

All but one of the 18 FGD participants (95%) agreed that the mild scarring was acceptable to them, likening it to a mosquito bite or a scar from a body piercing, and one participant (5%) was unsure. For many, this mild scarring was similar to their own scars, which they felt were acceptable, and would not draw attention.

*I’m the only one that would know of the minor changes I see*,* because it is my body; but another person wouldn’t notice anything here*. (Yvonne, FGD 1)

### Hyperpigmentation

Participants were also shown anonymised examples of the severe, moderate, and mild hyperpigmentation experienced by some trial participants and expressed concern that this could potentially expose women to stigma or misunderstanding.

Severe hyperpigmentation was regarded by 15 of the 18 FGD participants (84%) as being unacceptable due to its visibility, thereby drawing attention. The primary concern was that the discoloration could be associated with experiences of physical violence, which carried with it substantial social stigma.

*This looks hideous*,* it looks bad… People now pay attention to things. They might think you were beaten*,* or your partner abused you badly*,* so people will want to know the story. Some won’t believe that an implant can do this.* (Zahara, FGD 1)

*I also don’t like this. I wear short sleeves. This will make me seem like a victim of physical abuse with a bruise.* (Participant, FGD 2)

*Other people will not believe that this was left by the implant you inserted. It is like you were involved in some criminal activity and obtained an injury from it, which resulted in you having this big dark mark.* (Nonku, FGD 3)

In addition to the fear of social stigma resulting from the association between bruising or darkened skin and violence, participants also expressed that it would be difficult for them personally to accept the change to their own skin tone.

Mild hyperpigmentation was seen as acceptable by half of the participants, who thought it looked ‘normal’ and would fade with time. Others felt this would still draw too much attention or were not sure if it was acceptable.

*It is still bright and visible. I think you’d struggle to wear something with short sleeves in this situation because people will ask you questions*,* and you will have to keep telling your story to everyone…. Others won’t understand*,* and they’ll say you are lying*,* you are just hiding the fact that your man beats you up*,* because it is visible.* (Annie, FGD 3)

All FGD participants felt that the mild hyperpigmentation would be acceptable.

### Prior Warning of Potential Skin Reactions

Participants expressed differing views on how much should be shared with other women about the possible severe scars and hyperpigmentation from using a TAF implant, reflecting a tension between transparency about risks and concern for the social or emotional consequences for others. Some felt it was better to be upfront about this, to warn women about these potential effects.

*I think it’s better for you to explain to someone first what they can expect*,* and let them decide for themselves… Don’t hide things that can go wrong. Let them know*,* and they will choose for themselves.* (Nonu, FGD 3)

Others thought that this would set up unnecessary concerns for potential users, who might not themselves have similar severe reactions.

*If I had to tell another person about the implant*,* it would not be a good idea to show them my scar*,* because I don’t think anyone would agree to be left looking like this.* (Sli, FGD 3)

### Considering the Overall Value of the TAF Implant

During the FGDs, participants weighed the drawbacks of scarring and hyperpigmentation against the benefits of HIV prevention and practical advantages, describing the implant as worthwhile despite potential skin-related impact.

Many reflected that that long-acting nature of the implant with its reduced clinic visits, reduction in pill burden, likely improved PrEP adherence and discretion would motivate other women to use it.

*The Implant is Convenient,* *as you don’t have to go to the Clinic. (Luh, IDI)*

*When having an implant*,* you don’t forget about drinking pills. It’s always present and you never forget to apply it*,* since it is in you. (White*,* IDI)*

*Without having to be joined with another person… you know that you can protect yourself in the way you want*,* no matter what happens.* (Mimi, IDI)

Despite the concerns regarding scarring and hyperpigmentation, many participants were excited to be part of testing a new technology for HIV prevention, and expressed a desire for continued research into the implant for HIV prevention.

*I would like this TAF implant to succeed*,* so that women will be protected. Because its side-effects are avoidable*,* and you are the one who knows how to avoid them; I would like it to continue to work*,* because it is not that bad.* (Pinky, IDI)

## Discussion

Participants’ feelings about visibility, scarring, and hyperpigmentation were shaped by individual experiences, social interactions, and broader contextual factors, reflecting the multi-level influences emphasised in the Social Ecological Model [11]. Scars and changes in skin colouration are “written on the skin”, legible to others and interpreted through social and cultural lenses, shaping perceptions of acceptability. These findings suggest that perceptions of bodily changes are not experienced in isolation but are interpreted in light of anticipated social scrutiny, potential stigma, and the cultural and relational norms that surround the participants’ everyday lives.

Participants voiced that unwelcome comments and questions about the implant that might result from either the visibility of implant or the marks it left behind on the skin would be concerning for them. This echoes findings from research into experiences with the sub-dermal contraceptive implant, which suggests that women feel vulnerable to both intrusions on their privacy and the implant being violently removed by criminals if it is visible [12].

The range of responses to the ISRs experienced during the trial reflects mixed reactions, where some participants adopt a stoic approach and accept the side-effects as part of the trial process, while others express concern about potential long-term implications. These findings track with acceptability assessments done in the primary clinical trial [13]. Given the concerns that black South African women have about hyperpigmentation and scarring resulting from acne and the use of skin lighteners [14], these concerns about the impact of ISRs are unsurprising.

While most participants were not concerned by the scars and hyperpigmentation that remained on their own skin after implant removal and exiting the clinical trial, they were disturbed by the photographs shown of moderate or severe scarring and discoloration experienced by other participants. Scars and hyperpigmentation are often perceived as being unsightly and painful, causing distress for women who would prefer that these are not visible to others [15]. Scarring and dark marks are also often associated with disease, dirt or unattractiveness [16] and the participants reiterated these associations.

Beyond concerns for their own skin and sense of healthiness or attractiveness, participants were more concerned that scars and marks would draw attention that would result in them experiencing social stigma. These fears linked to stigma related to constructs around experiencing violence and around women’s’ sexual behaviour. Women in South Africa who have survived violence (particularly rape or sexual violence) often experience both external stigma from members of society who see them as devalued or discredited [17] and internal stigma with high levels of shame and self-blame [18]. Given the belittling social attitudes about women who have experienced violence, it is unsurprising that participants did not want scars or discolouration from the implant to be misconstrued as scars and bruising from violence.

There are also high levels of stigma around contraception use in South Africa, with a social cost associated with women (particularly younger women) acknowledging that they are sexually active and taking action to prevent pregnancy [19]. The trial participants did not want their TAF implant scars to be mis-identified as contraceptive implant scars, which they thought a likely response, as there is wider public knowledge about the implants for contraception.

Most participants were reassured at their lack of severe scars or hyperpigmentation after the implant was removed, reporting a sense of relief from both physical discomfort and anxiety about potential long-term damage to the skin. Where participants had been badly scarred themselves, they noted this took time to get used to, and had negatively changed the way they dressed and impacted on their self-confidence.

The high prevalence of scarring and hyperpigmentation during the trial highlights the need for biomarkers or other predictors of moderate to severe skin reactions or low tolerability to implant insertion. Such tools could enable the identification of individuals at higher risk of ISRs prior to trial enrolment, supporting more informed decision-making and allowing clinicians to proactively manage side effects; ultimately reducing the likelihood of complications or early discontinuation.

### Limitations

While this study included a substantial proportion of the total participant population (*n* = 29/36), the small number of participants limits statistical generalisability. Our findings reflect interpretive insights into shared experiences rather than population-level conclusions. Despite reflexive engagement and transparent coding, the interpretation remains shaped by the analyst’s perspective.

## Conclusions

While recognising the potential for scarring and discolouration caused by the implant, with the resultant self-consciousness, negative judgments and related social stigma, participants felt that that the implant was preferrable to other PrEP technologies. Participants generally expressed positive views about this type of technology, and considered that other women would be interested in adopting it should it be effective in preventing HIV.

Continued research with an improved formulations that enhance drug release rates should consider how best to minimise the severity of ISRs and visible skin changes, for a more acceptable implant that women would take up without concern for their appearance and the stigma that marks leave on their skin.

## Data Availability

Anonymised study data sets may be made available to investigators whose proposed use of the data has been approved by the CAPRISA Scientific Review Committee. Requests to access the data can be made through the CAPRISA website (www.caprisa.org).
